# Estimation of bed net coverage indicators in Tanzania using mobile phone surveys: a comparison of sampling approaches

**DOI:** 10.1186/s12936-022-04408-y

**Published:** 2022-12-10

**Authors:** Matt Worges, Benjamin Kamala, Joshua Yukich, Frank Chacky, Samwel Lazaro, Charles Dismas, Sijenun Aroun, Raya Ibrahim, Mwinyi Khamis, Mponeja P. Gitanya, Deodatus Mwingizi, Hannah Metcalfe, Willhard Bantanuka, Sena Deku, David Dadi, Naomi Serbantez, Dana Loll, Hannah Koenker

**Affiliations:** 1 USAID Tanzania Vector Control Activity, Tropical Health, New Orleans, LA USA; 2 USAID Tanzania Vector Control Activity, Johns Hopkins University School of Public Health Center for Communication Programs, Dar Es Salaam, Tanzania; 3 Tanzania National Malaria Control Program, Dodoma, Tanzania; 4 Zanzibar Malaria Elimination Programme, Zanzibar, Tanzania; 5 Viamo, Dar Es Salaam, Tanzania; 6 U.S. President’s Malaria Initiative, Dar Es Salaam, Tanzania; 7 USAID Tanzania Vector Control Activity, Johns Hopkins University School of Public Health Center for Communication Programs, Baltimore, MD USA; 8 USAID Tanzania Vector Control Activity, Tropical Health, Baltimore, MD USA

**Keywords:** ITN, Mobile phone survey, Random digit dial, Coverage indicators, Tanzania

## Abstract

**Background:**

Threats to maintaining high population access with effective bed nets persist due to errors in quantification, bed net wear and tear, and inefficiencies in distribution activities. Monitoring bed net coverage is therefore critical, but usually occurs every 2–3 years through expensive, large-scale household surveys. Mobile phone-based survey methodologies are emerging as an alternative to household surveys and can provide rapid estimates of coverage, however, little research on varied sampling approaches has been conducted in sub-Saharan Africa.

**Methods:**

A nationally and regionally representative cross-sectional mobile phone survey was conducted in early 2021 in Tanzania with focus on bed net ownership and access. Half the target sample was contacted through a random digit dial methodology (n = 3500) and the remaining half was reached through a voluntary opt-in respondent pool (n = 3500). Both sampling approaches used an interactive voice response survey. Standard RBM-MERG bed net indicators and AAPOR call metrics were calculated. In addition, the results of the two sampling approaches were compared.

**Results:**

Population access (i.e., the percent of the population that could sleep under a bed net, assuming one bed net per two people) varied from a regionally adjusted low of 48.1% (Katavi) to a high of 65.5% (Dodoma). The adjusted percent of households that had a least one bed net ranged from 54.8% (Pemba) to 75.5% (Dodoma); the adjusted percent of households with at least one bed net per 2 de facto household population ranged from 35.9% (Manyara) to 55.7% (Dodoma). The estimates produced by both sampling approaches were generally similar, differing by only a few percentage points. An analysis of differences between estimates generated from the two sampling approaches showed minimal bias when considering variation across the indicator for households with at least one bed net per two de facto household population.

**Conclusion:**

The results generated by this survey show that overall bed net access in the country appears to be lower than target thresholds. The results suggest that bed net distribution is needed in large sections of the country to ensure that coverage levels remain high enough to sustain protection against malaria for the population.

**Supplementary Information:**

The online version contains supplementary material available at 10.1186/s12936-022-04408-y.

## Background

Malaria is a major public health issue and it is estimated that 229 million cases occurred globally in 2019, with Africa experiencing the largest burden [1]. It is endemic throughout most of mainland Tanzania and in Zanzibar [1, 2], and is a major cause of morbidity and mortality among children under five years of age and pregnant women [1]. According to Tanzania Demographic and Health Surveys (DHS), prevalence of malaria in children under five years of age declined by half from 18% in 2007–08 to 9% in 2011–12 [3, 4]. The 2017 Malaria Indicator Survey (MIS) data show a reduction in malaria prevalence from about 14% in 2015–16 to 7.5%; however, malaria parasite prevalence demonstrates a high degree of regional variation ranging from 24.4% to near zero [2, 5].

Due to the high prevalence and burden of malaria in Tanzania, the National Malaria Control Programme (NMCP) and the Zanzibar Malaria Elimination Programme (ZAMEP), in collaboration with their partners, implement several recommended preventive and curative interventions. One of the primary preventive strategies for reducing malaria transmission is the distribution and use of insecticide-treated bed nets (ITNs). Bed nets have been responsible for an estimated 68% reduction in global malaria cases since 2000 [6].

In Tanzania, the continuing efforts of the NMCP, ZAMEP, and their partners have achieved and largely sustained high population access to ITNs over the past several years. Globally, malaria control strategies are moving towards stratification by malaria prevalence to improve targeting of interventions and further reduce transmission and risk. Tanzania is at the forefront of this movement, having stratified its approaches at a council level in the latest National Strategy [7]. This National Strategy calls for a blend of ITN distribution channels depending on a council’s designated stratum with low, moderate, and high transmission settings delivering ITNs through annual school distributions, at reproductive and child health services (first antenatal clinic and immunization visits, respectively), and in mass campaigns when necessary. In very-low transmission strata, only ITN distribution via reproductive and child health services is implemented, and in urban locales ITNs are expected to be available in the commercial sector. The 2020 mass replacement campaign was implemented in 50 districts across 10 regions of mainland Tanzania; 14 regions of the mainland have implemented annual school distributions for the past 5–8 years, supported by the President’s Malaria Initiative, increasing the need for annual monitoring to inform quantifications for the subsequent year to maintain ITN access at high levels. Zanzibar has implemented a combination of mass campaigns, antenatal care distribution, and community-based distribution. However, threats to maintaining high population access with effective bed nets persist. Decreased bed net access resulting, in part, from errors in quantification, bed net wear and tear, and inefficiencies in distribution activities requires monitoring and surveillance to adapt and respond with appropriate interventions [2, 8].

Monitoring and surveillance of bed net distribution and coverage typically rely on household surveys, which are expensive, time consuming, and infrequent [9]. Alternatives such as lot quality assurance sampling surveys have been used in Tanzania and elsewhere, but such approaches are still household based and require significant investments in logistics and transportation [10–13]. In addition, given the COVID-19 pandemic, it was preferable to collect data using a method that eliminates face-to-face contact such as mobile phone surveys, which can protect both the participant and the interviewer.

Due to improvements in the affordability of mobile technology and ongoing network deployments to increase coverage areas, mobile phone ownership in low- and middle-income countries (LMIC) has rapidly increased [14–18]. This growth has resulted in mobile phone-based survey methodologies emerging as a comparatively inexpensive alternative to large scale population-based household surveys [19–23]. In LMIC settings, the use of random digit dialing (RDD) presents as an increasingly viable mobile phone survey option [24–26]; however, little research on varied sampling approaches has been conducted in sub-Saharan Africa.

A previous interactive voice response (IVR) random digit dial (RDD) mobile phone survey was conducted in Tanzania immediately following the 2017 MIS (unpublished data). The study demonstrated that while the degree of regional concordance between the RDD mobile phone survey and the 2017 MIS varied by assessed bed net indicator, generally the minimum, median, and maximum values for each indicator were consistent, suggesting that RDD mobile phone surveys are an acceptable, lower cost option for monitoring bed net coverage in Tanzania. The current study used a mobile phone survey with a dual sampling approach to provide cross-sectional estimates of bed net ownership and access across regions of mainland Tanzania and Zanzibar. This study also provided an opportunity to assess any apparent bias and efficiency gains in sampling from a frame of opt-in survey participants versus the standard RDD approach.

## Methods

Viamo, the mobile phone survey operator, conducted the nationally and regionally representative cross-sectional mobile phone survey in Tanzania using an IVR method. The survey ran from January to March of 2021. Half the target was planned to be reached through a standard RDD methodology and the remaining half was planned to be reached by sampling from a voluntary opt-in respondent pool to allow for comparison of the two approaches. For both sampling approaches, Viamo programmed pre-recorded voice instructions in Kiswahili and respondents used the numeric keypad to input responses to a brief set of questions. Briefly, survey questions covered region of residence (for RDD respondents), household member counts, household bed net counts, and durable goods ownership (see Additional File 1 for a complete list survey questions). Questions for durable goods ownership were used in a poststratification weighting process to reduce the sampling error and potential non-response bias. Ineligible respondents were those under the age of 18 years or whose call was ended as the sample quota had been reached.

### RDD sampling approach

For the RDD approach, Viamo dialed phone numbers at random to generate a pool of potential participants. Tanzania has a 12-digit mobile number structure with a prefix of 255 (the country code) followed by a three-digit secondary prefix of 51 different possibilities. From among the 51 different trunk number sequences, the final six digits are those that were randomly generated one at a time for each call.

### Opt-in respondent pool sampling approach

The mobile network operator, Vodacom, shared with Viamo an ‘opt-in’ database of Tanzania-based phone numbers for respondents who reported that they would be willing to participate in mobile phone surveys related to health and well-being. The ‘opt-in’ database includes information on the region of residence of all individuals thereby reducing the cascade-style questions necessary to obtain such information. In utilizing the opt-in survey approach, the call center randomly sampled from geographic zone-specific blocks of numbers until regional quotas were met. The date that a Vodacom subscriber opted into the survey program is included in the opt-in database and Viamo was able to prioritize phone numbers from participants who had opted into the survey program within the 12 months preceding the survey. In the event there was difficulty reaching targeted sample sizes, the mobile phone survey operator was able to sample from older participant rolls.

### Outcomes

In addition to project-specific indicators of interest, the primary outcomes for this monitoring activity include the following Roll Back Malaria Partnership to End Malaria-Monitoring and Evaluation Reference Group (RBM-MERG) indicators [27]:

1. Percent of households with at least one bed net of any type
2. Percent of households with at least one bed net of any type for every two people
3. Percent of de facto household population with access to a bed net of any type within their household.

Two non-RBM-MERG bed net indicators were also calculated:

1. Percent of bed nets self-reported by respondents as purchased
2. Percent of bed nets self-reported by respondents as originally or ever having been treated with insecticide

The non-RBM-MERG indicators were calculated at the bed net level. All bed net indicators were estimated by region and separately for the entirety of mainland Tanzania and Zanzibar.

### Definitions

The American Association for Public Opinion Research (AAPOR) asks that mobile phone survey practitioners use AAPOR standard definitions for comparability across studies [28]. As such, standard definitions were used for the contact (CON3), response (RR5 and RR6), cooperation (COOP1 and COOP2), and refusal/break-off (REF3) rates for each sampling strategy. These measures are defined below where I = complete interview, P = partial interview, R = refusal and break-off, NC = non-contact, and O = other. Table 1 below describes the AAPOR call dispositions used to classify respondent calls.

**Table 1:** Call disposition descriptions

Disposition	Description
Complete	Complete interview designations required that participants answer the questions necessary for calculation of the bed net indicators of interest as well as those for poststratification adjustment (i.e., bicycle, TV, and radio ownership)
Partial	Partial interview designations only required that participants answer the questions necessary for calculation of the bed net indicators of interest, but not those used for poststratification adjustment
Breakoff	Breakoff interview designations denote those interviews in which a participant started answering questions, but ultimately broke off the call before a designation of complete or partial could be established
Refusal	Refusals were characterized by respondents who indicated that they were not interested in participating in the survey
Non-contact	Non-contact interviews are those in which the participant could not be reached (no answer or non-assigned phone number)
Other	If an interview was designated as anything other than complete, partial, breakoff, or refusal, it was dispositioned as ‘other’. In most instances, a designation of ‘other’ resulted from skipped questions necessary for a disposition of complete/partial, but in which the respondent finished the survey
Ineligible*	Ineligible respondents are those who were under the age of 18 years or those whose call was ended as the sample quota had been reached for their region
Finished*	Finished interviews was used as an internal program designation and was used to track progress towards sample size goals. This would include calls dispositioned as complete, partial, and other so long as the participant reached the end of the survey. As such, while this designation was used to track progress towards sample size goals, this category of interviews is not meaningful from an analytic perspective

^*^Not an official AAPOR disposition category: the AAPOR provides guidance on dispositioning calls but encourages researchers to establish a priori definitions of what constitutes a complete vs. a partial interview and what distinguishes a partial interview from a break-off. Otherwise, prescribed equations are provided for calculating rates (i.e., response, cooperation, refusal, and contact rates) using call dispositions. Note that regardless of call disposition status, the RBM-MERG bed net coverage indicators were calculated if the requisite data elements were available

$$CON3=\frac{\left(I+P\right)+R+O}{\left(I+P\right)+R+O+NC}$$

$$RR5= \frac{I}{\left(I+P\right)+\left(R+NC+O\right)}$$

$$RR6= \frac{I+P}{\left(I+P\right)+\left(R+NC+O\right)}$$

$$COOP1=\frac{I}{\left(I+P\right)+R+O}$$

$$COOP2=\frac{(I+P)}{\left(I+P\right)+R+O}$$

$$REF3=\frac{R}{\left(I+P\right)+\left(R+NC+O\right)}$$

Based on the call disposition categories described in Table 1, the number of finished interviews is higher than the number of complete/partial interviews, and complete interviews, being more restrictive in their designation, are a subset of partial interviews. All available data points were taken into consideration for calculation of each individual indicator of interest regardless of call disposition status, which resulted in differing sample sizes across indicators for the same regions.

### Study size

For the IVR mobile phone survey, it was estimated that the precision of the estimate within each region should be similar to the precision obtained in a DHS or MIS. In a recent Tanzania DHS (2015–2016) and MIS (2017), approximately 400 households were chosen in approximately 20 enumeration areas per region. Assuming an 80% baseline ownership of at least one bed net at the household level a sample of 440 households in a cluster sample with a design effect of 2 has approximately 80% power to detect a difference of seven percentage points. This means that a mobile phone survey with a regional sample size of approximately 250 households should yield a similar precision assuming simple random sampling. The regional sample size target was split between two contact/sampling methods with a goal of 125 finished surveys per region from both the RDD and opt-in methods described above. Given the 28 regions in Tanzania (26 in mainland Tanzania plus Unguja and Pemba as two regions of Zanzibar), the total sample size is 7,000 surveys with 3,500 surveys coming from each sampling approach. For both sampling approaches, potential participants were recruited until regional quotas were met.

### Data analysis

A number of quality checks were built into the survey such that participants who provided illogical responses (e.g., reporting more people sleeping in the household than household members, reporting more insecticide-treated bed nets than total bed nets in the household) heard an error message and were prompted to re-enter a valid value. Unweighted/unadjusted estimates were calculated for all indicators of interest. Following the initial analysis**,** poststratification adjustment was used to weight regional bed net indicator estimates. The marginal proportions of radio, TV, and bicycle ownership as well as region were calculated from the 2017 MIS and corresponding attributes from the combined RDD/opt-in surveys were raked to these MIS marginal proportions. Including region in the raking process ensured poststratification adjustment by the necessary administrative unit. All values in the narrative component of the report present adjusted indicator estimates. Additionally, logistic regression analyses were run for the two household-level RBM-MERG indicators with survey approach (RDD vs opt-in) as the outcome variable and household-level bed net indicator values used as predictor variables. Each regression controlled for region, radio and bicycle ownership, and household size.

## Results

A total of 310,151 calls were placed to 246,233 unique phone numbers (Table 2). Of the total calls placed, 163,748 contacts were made (including refusals & breakoffs, partial/complete interviews, other classifications, and ineligible respondents – see Table 1 for a description of call dispositions). Of the 163,748 contacts made, 6,968 participants made it to the end of the survey regardless of whether they answered each question along the way (i.e., they may have skipped certain questions, but still finished the survey). A total of 3,020 interviews were designated as complete, meaning all bed net indicators of interest for these respondents could be calculated and for which poststratification adjustment could be conducted. Interviews designated as ‘complete’ were used to calculate overall response rate. The percent of finished interviews varied by region from 177% of target (Unguja region; n = 443) to 48% of target (Pemba region; n = 121).

**Table 2:** Call characteristics and AAPOR call disposition by method

	Combined methods	RDD method only	Opt-in method only
Calls			
Total calls placed	310,151	142,946	167,205
Total unique numbers called	246,233 (79.4%)	138,728 (97.0%)	107,505 (64.3%)
Call disposition			
(NC) Non-contacts	82,485 (33.5%)	55,673 (40.1%)	26,812 (24.9%)
(R) Refusals & break-offs	127,375 (51.7%)	74,741 (53.9%)	52,634 (49.0%)
(P) Partial interviews	3,192 (1.3%)	1,551 (1.1%)	1,641 (1.5%)
(I) Completed interviews	3,020 (1.2%)	1,462 (1.1%)	1,558 (1.4%)
(O) Other	5,448 (2.2%)	2,053 (1.5%)	3,395 (3.2%)
Ineligible respondents†	27,733 (11.3%)	4,710 (3.4%)	23,023 (21.4%)
AAPOR designations			
Contact rate 3 (*CON3*): (I+P)+R+O/(I+P)+R+NC+O	62.2%	58.5%	68.3%
Response rate 5 (*RR5*): I/((I+P)+(R+NC+O))	1.4%	1.1%	1.8%
Response rate 6 (*RR6*): (I+P)/((I+P)+(R+NC+O))	1.5%	1.2%	1.9%
Cooperation rate 1 (*COOP1*): I/(I+P) + R+O)	2.2%	1.9%	2.7%
Cooperation rate 2 (*COOP2*): (I+P)/((I+P)+R+O))	2.3%	2.0%	2.8%
Refusal rate 3 (*R3*): R/((I+P)+(R+NC+O))	58.3%	55.8%	62.3%
Survey Details			
Average survey length (I + P)	9 min 51 s	9 min 49 s	9 min 52 s

*RDD* random digit dial; *AAPOR:* American Association of Public Opinion Research *min* minutes; *sec* seconds

^†^Not an official AAPOR designation—ineligible respondents were under the age of 18 years or were excluded from participation because their call exceeded the regional quota

The response rate was approximately 1.5% across the combined RDD and opt-in survey methodologies. Because the opt-in method was expected to have fewer non-contact calls by virtue of the opt-in nature of the program, the cooperation rate, defined in Table 2, can be assessed to get an alternative indication of successful interviews as non-contacts are removed from the denominator. This metric was calculated at 2.7% for complete interviews. Table 2 also presents call characteristics by survey method (RDD and opt-in). Nearly one-quarter (24.9%) of calls placed to the opt-in group were designated as non-contacts. The cooperation rates between the two methods differed by about 0.8 percentage points in favor of the opt-in method. The average length of surveys with a call disposition of complete or partial was just under ten minutes. Just over three-quarters (77.7%) of respondents with complete/partial call designations finished the survey within 7.5 to 11.5 min.

Because respondents could skip or refuse to answer any question, the desired sample size of 250 respondents per region was not achieved for all assessed indicators despite reaching 99.5% of the overall target of finished interviews (6,968 of 7,000). The indicator for households with at least one bed net of any type had at least 250 valid responses for all regions except for Pemba where the sample size was 213 households or 81.5% of the target. For the other two assessed RBM-MERG indicators (percent of households with at least one bed net of any type for every two people and percent of de facto household population with access to a bed net of any type within their household), the desired sample size of 250 households was not reached for Katavi, Lindi, Mtwara, Rukwa, and Pemba regions. Aside from Pemba, which only reached 48% of the target, each of the aforementioned regions was within approximately 75% of the target. Additional File 2 shows the number of observations available by region to calculate assessed indicators.

The results for the three assessed RBM-MERG indicators are shown in Table 3. Population access to a bed net varied from an adjusted low of 48.1% in Katavi region to an adjusted high of 65.5% in Dodoma region. The adjusted percent of households that had a least one bed net ranged from 54.8% (Pemba) to 75.5% (Dodoma); the adjusted percent of households with at least one bed net per 2 de facto household population ranged from 35.9% (Manyara) to 55.7% (Dodoma); and the de facto household population access to a bed net ranged from an adjusted percent of 48.1% (Katavi) to 65.5% (Dodoma). Unweighted estimates are generally lower than those produced from the poststratification process for households with at least one bed net. The average regional difference between the two estimations is + 2.6 percentage points for this indicator although six regions exceed a difference of + 5 percentage points. Three-quarters of the regional estimates for both households with at least one bed net per two de facto household population and population access to a bed net were within ± 4 percentage points across the two estimates. Overall, adjusted regional estimates for households with at least one bed net per two de facto household population were generally lower than the unweighted estimates whereas the opposite is true of population access to a bed net.

**Table 3:** Unweighted and adjusted RBM-MERG bed net indicator estimates by region

	Household has 1 + bed net			Household has 1 + bed net per 2 de facto population			De facto population access to a bed net		
	Unweighted†	Adjusted*	% pt. diff	Unweighted†	Adjusted*	% pt. diff	Unweighted†	Adjusted*	% pt. diff
	% [95% CI]	% [95% CI]		% [95% CI]	% [95% CI]		% [95% CI]	% [95% CI]	
Mainland	65.5 [64.6, 66.3]	68.4 [67.1, 69.7]	2.9	50.5 [49.4, 51.5]	48.1 [46.5, 49.6]	−2.4	56.2 [55.0, 57.3]	57.3 [55.6, 59.0]	1.1
Zanzibar	53.0 [49.7, 56.2]	55.4 [49.7, 61.0]	2.4	42.4 [38.2, 46.7]	43.8 [36.7, 51.0]	1.4	44.9 [40.0, 49.8]	49.1 [40.9, 57.2]	4.2
Arusha	56.1 [52.2, 60.1]	61.6 [55.4, 67.9]	5.5	38.2 [33.8, 42.7]	36.6 [30.0, 43.3]	−1.6	45.7 [43.9, 47.5]	48.7 [41.8, 55.5]	3.0
Dar es salaam	68.9 [65.1, 72.6]	70.4 [64.4, 76.4]	1.6	53.9 [49.2, 58.7]	50.4 [43.1, 57.6]	−3.6	63.3 [61.5, 65.1]	62.0 [53.9, 70.1]	−1.4
Dodoma	69.8 [65.9, 73.7]	75.5 [69.7, 81.3]	5.7	58.1 [53.2, 62.9]	55.7 [48.4, 63.1]	−2.3	63.4 [61.6, 65.3]	65.5 [57.9, 73.2]	2.1
Geita	65.9 [61.8, 70.1]	73.5 [67.7, 79.3]	7.5	49.1 [43.9, 54.4]	46.1 [38.8, 53.3]	−3.1	54.9 [53.0, 56.9]	55.2 [47.0, 63.3]	0.2
Iringa	72.6 [68.9, 76.3]	78.4 [73.1, 83.8]	5.8	58.0 [53.4, 62.7]	55.4 [48.5, 62.4]	−2.6	60.1 [58.2, 62.0]	64.9 [57.4, 72.4]	4.8
Kagera	68.9 [65.0, 72.8]	67.9 [62.0, 73.7]	−1.1	52.2 [47.4, 57.1]	49.2 [42.1, 56.3]	−3.0	59.4 [57.4, 61.3]	59.9 [52.2, 67.6]	0.5
Katavi	60.0 [54.4, 65.6]	56.9 [48.1, 65.7]	−3.1	47.0 [39.8, 54.2]	46.6 [35.7, 57.4]	−0.5	51.1 [48.4, 53.7]	48.1 [36.9, 59.4]	−2.9
Kigoma	63.0 [58.5, 67.6]	70.4 [63.8, 77.1]	7.4	49.1 [43.2, 55.0]	52.5 [44.2, 60.8]	3.4	55.7 [53.4, 58.0]	62.2 [54.0, 70.5]	6.6
Kilimanjaro	60.8 [56.7, 65.0]	64.5 [58.3, 70.7]	3.7	44.8 [40.0, 49.7]	40.2 [33.3, 47.2]	−4.6	48.4 [46.4, 50.3]	50.3 [43.1, 57.6]	2.0
Lindi	67.0 [61.8, 72.2]	68.6 [60.3, 77.0]	1.7	55.7 [48.8, 62.5]	55.6 [44.7, 66.5]	−0.1	61.3 [58.9, 63.7]	60.7 [48.3, 73.1]	−0.6
Manyara	60.2 [55.2, 65.2]	60.1 [51.9, 68.3]	−0.1	46.8 [40.7, 53.0]	35.9 [26.6, 45.2]	−10.9	50.3 [48.0, 52.6]	51.1 [40.1, 62.2]	0.8
Mara	69.8 [65.5, 74.0]	71.7 [65.8, 77.7]	2.0	46.8 [41.6, 52.1]	39.7 [32.8, 46.7]	−7.1	57.8 [55.9, 59.7]	56.4 [49.5, 63.4]	−1.4
Mbeya	67.8 [63.7, 71.8]	73.1 [66.9, 79.3]	5.4	53.8 [49.0, 58.7]	51.0 [43.3, 58.7]	−2.8	55.8 [53.8, 57.8]	57.0 [48.8, 65.2]	1.2
Morogoro	64.6 [60.5, 68.7]	65.1 [58.7, 71.4]	0.5	53.8 [48.6, 58.9]	55.2 [47.3, 63.2]	1.5	58.9 [56.8, 60.9]	56.9 [47.3, 66.4]	−2.0
Mtwara	64.0 [58.4, 69.5]	68.5 [59.8, 77.2]	4.5	49.2 [42.1, 56.3]	52.4 [41.5, 63.3]	3.2	51.4 [48.6, 54.2]	56.6 [45.1, 68.1]	5.2
Mwanza	68.4 [64.7, 72.0]	72.1 [66.6, 77.5]	3.7	53.0 [48.4, 57.7]	55.3 [48.6, 62.1]	2.3	60.9 [59.1, 62.6]	63.6 [56.2, 71.1]	2.8
Njombe	60.7 [55.8, 65.6]	63.0 [55.6, 70.4]	2.2	48.0 [42.4, 53.6]	51.4 [43.2, 59.6]	3.4	52.6 [50.1, 55.0]	59.2 [49.9, 68.4]	6.6
Pwani	72.3 [67.8, 76.8]	76.3 [69.4, 83.2]	4.0	56.9 [51.1, 62.8]	52.9 [43.9, 61.9]	−4.1	62.7 [60.4, 65.0]	64.2 [55.4, 73.0]	1.5
Rukwa	62.0 [56.8, 67.3]	63.2 [54.3, 72.0]	1.1	46.5 [39.9, 53.2]	46.1 [35.1, 57.1]	−0.4	49.7 [47.2, 52.2]	56.6 [45.7, 67.6]	6.9
Ruvuma	68.9 [64.8, 73.1]	72.2 [66.1, 78.3]	3.3	53.4 [48.2, 58.7]	50.4 [43.0, 57.9]	−3.0	58.3 [56.2, 60.4]	60.0 [51.8, 68.1]	1.7
Shinyanga	64.4 [60.1, 68.7]	65.3 [58.7, 71.9]	0.9	48.7 [43.4, 54.0]	42.5 [34.4, 50.5]	−6.2	57.2 [55.1, 59.3]	53.4 [45.0, 61.8]	−3.8
Simiyu	65.2 [60.5, 69.9]	68.0 [61.2, 74.9]	2.8	40.8 [35.0, 46.6]	37.8 [29.6, 45.9]	−3.0	51.3 [49.2, 53.3]	51.8 [43.6, 59.9]	0.5
Singida	66.3 [62.2, 70.4]	68.0 [61.3, 74.7]	1.7	53.0 [47.8, 58.2]	50.8 [42.7, 58.9]	−2.2	56.3 [54.4, 58.3]	57.4 [48.2, 66.7]	1.1
Songwe	58.4 [53.4, 63.3]	59.2 [51.1, 67.3]	0.8	47.3 [41.2, 53.4]	51.1 [41.8, 60.3]	3.8	48.2 [45.8, 50.5]	51.0 [40.1, 61.9]	2.8
Tabora	63.8 [59.7, 67.8]	67.0 [60.7, 73.4]	3.3	47.2 [42.1, 52.2]	43.4 [35.6, 51.2]	−3.8	57.2 [55.3, 59.0]	59.1 [51.1, 67.1]	1.9
Tanga	66.7 [62.6, 70.8]	66.2 [59.5, 72.9]	−0.5	55.8 [50.7, 61.0]	55.3 [47.2, 63.5]	−0.5	59.6 [57.7, 61.4]	59.6 [50.4, 68.8]	0.0
Pemba	55.9 [49.2, 62.5]	54.8 [43.2, 66.4]	−1.1	45.5 [36.6, 54.3]	48.4 [33.6, 63.1]	2.9	54.6 [51.3, 58.0]	50.3 [33.2, 67.4]	−4.3
Unguja	52.1 [48.4, 55.8]	55.6 [49.1, 62.1]	3.5	41.5 [36.7, 46.4]	42.6 [34.4, 50.7]	1.1	41.9 [40.1, 43.8]	48.8 [39.6, 58.1]	6.9

^†^Calculated from all available data points regardless of the presence of values for variables used in the poststratification process

^*^Only records with available data points for poststratification adjustment could be used to produced adjusted estimates. As such, adjusted estimates are calculated from a truncated data set compared to the data set used to calculate the unweighted estimates

*CI* confidence interval; *% pt. diff.* percentage point difference

Additional non-RBM MERG indicators were also calculated including the percent of bed nets self-reported by survey respondents as purchased from an adjusted low of 18.9% (Songwe) to an adjusted high of 59.8% (Arusha) (Table 4). The indicator for the percent of bed nets self-reported by survey respondents to have ever been treated with insecticide which ranged from an adjusted low of 24.0% (Ruvuma) to an adjusted high of 56.2% (Mbeya).

**Table 4:** Non-RBM-MERG bed net indicators by region

	Bed nets self-reported as purchased			Bed nets self-reported as originally or ever treated with insecticide		
	Unweighted†	Adjusted*	% pt. diff	Unweighted†	Adjusted*	% pt. diff
	% [95% CI]	% [95% CI]		% [95% CI]	% [95% CI]	
Mainland	38.4 [36.8, 39.9]	38.9 [36.8, 41.0]	−0.5	42.3 [40.6, 43.9]	38.6 [36.2, 41.0]	−3.7
Zanzibar	40.1 [33.0, 47.1]	46.8 [35.6, 58.0]	+ 6.7	41.1 [34.4, 47.8]	42.8 [32.3, 53.4]	+ 1.7
Arusha	51.4 [48.5, 54.3]	59.8 [50.5, 69.0]	+ 8.4	37.0 [34.2, 39.7]	31.1 [21.5, 40.8]	−5.8
Dar es salaam	48.5 [45.9, 51.2]	52.6 [42.5, 62.7]	+ 4.1	40.6 [38.1, 43.1]	37.0 [27.2, 46.8]	−3.6
Dodoma	38.4 [35.7, 41.1]	40.9 [31.2, 50.5]	+ 2.4	49.3 [46.5, 52.2]	49.9 [38.2, 61.6]	+ 0.5
Geita	34.2 [31.5, 37.0]	34.3 [26.0, 42.6]	+ 0.0	41.3 [38.4, 44.1]	32.1 [22.6, 41.6]	−9.2
Iringa	35.2 [32.6, 37.7]	31.5 [23.3, 39.7]	−3.7	53.0 [50.3, 55.7]	55.2 [44.3, 66.2]	+ 2.2
Kagera	24.1 [21.8, 26.4]	25.6 [17.6, 33.6]	+ 1.5	34.9 [32.2, 37.6]	28.1 [18.4, 37.7]	−6.8
Katavi	42.0 [38.2, 45.8]	53.4 [39.4, 67.3]	+ 11.3	37.9 [34.1, 41.6]	28.8 [13.2, 44.5]	−9.0
Kigoma	33.9 [30.8, 37.0]	42.4 [30.2, 54.5]	+ 8.5	40.0 [36.8, 43.3]	40.5 [28.5, 52.5]	+ 0.4
Kilimanjaro	39.2 [36.3, 42.0]	36.0 [26.0, 46.1]	−3.1	48.9 [45.9, 51.9]	33.7 [24.1, 43.4]	−15.2
Lindi	47.0 [43.7, 50.4]	48.6 [35.6, 61.6]	+ 1.6	45.2 [42.0, 48.4]	46.2 [31.4, 61.0]	+ 1.0
Manyara	36.7 [33.4, 39.9]	37.4 [24.1, 50.7]	+ 0.7	46.2 [43.0, 49.5]	39.3 [22.6, 55.9]	−7.0
Mara	40.7 [37.9, 43.4]	39.1 [29.7, 48.4]	−1.6	45.8 [42.9, 48.6]	46.4 [36.4, 56.4]	+ 0.6
Mbeya	31.8 [28.9, 34.7]	39.1 [30.5, 47.8]	+ 7.3	47.2 [44.0, 50.5]	56.2 [45.8, 66.7]	+ 9.0
Morogoro	46.9 [44.1, 49.7]	45.8 [33.9, 57.8]	−1.1	43.2 [40.5, 45.8]	41.8 [29.6, 54.0]	−1.4
Mtwara	39.5 [35.7, 43.2]	44.1 [29.7, 58.5]	+ 4.6	41.7 [38.1, 45.3]	39.8 [24.2, 55.4]	−1.9
Mwanza	33.8 [31.5, 36.1]	30.7 [23.3, 38.1]	−3.1	38.1 [35.6, 40.5]	35.5 [26.3, 44.8]	−2.5
Njombe	32.9 [29.5, 36.3]	32.8 [22.8, 42.9]	−0.0	40.5 [36.8, 44.1]	42.6 [28.7, 56.5]	+ 2.1
Pwani	46.9 [43.7, 50.2]	51.6 [39.3, 63.9]	+ 4.7	43.1 [39.9, 46.4]	33.2 [20.8, 45.5]	−10.0
Rukwa	33.3 [30.0, 36.7]	32.9 [21.9, 43.9]	−0.4	35.4 [32.0, 38.9]	38.8 [23.3, 54.4]	+ 3.4
Ruvuma	41.5 [38.5, 44.4]	39.5 [29.0, 49.9]	−2.0	32.8 [30.0, 35.6]	24.0 [15.7, 32.2]	−8.9
Shinyanga	45.1 [42.2, 48.1]	48.8 [37.3, 60.3]	+ 3.6	33.8 [31.1, 36.4]	33.8 [23.4, 44.1]	+ 0.0
Simiyu	39.9 [36.5, 43.2]	39.6 [28.5, 50.7]	−0.2	40.7 [37.7, 43.8]	37.4 [25.9, 48.8]	−3.4
Singida	35.9 [33.3, 38.5]	30.5 [20.5, 40.6]	−5.4	45.4 [42.6, 48.1]	39.2 [25.7, 52.7]	−6.2
Songwe	24.0 [21.2, 26.8]	18.9 [10.5, 27.2]	−5.1	39.0 [35.8, 42.2]	39.3 [24.6, 53.9]	+ 0.3
Tabora	35.7 [33.3, 38.2]	37.9 [28.9, 46.8]	+ 2.1	45.3 [42.7, 47.9]	38.7 [27.6, 49.7]	−6.6
Tanga	39.7 [37.0, 42.3]	42.7 [30.9, 54.4]	+ 3.0	53.8 [51.1, 56.5]	43.6 [31.8, 55.4]	−10.2
Pemba	39.6 [35.5, 43.7]	49.1 [26.8, 71.4]	+ 9.5	42.8 [38.7, 46.9]	36.0 [15.9, 56.0]	−6.8
Unguja	40.3 [37.6, 42.9]	45.7 [33.0, 58.5]	+ 5.5	40.6 [38.0, 43.1]	45.6 [33.4, 57.7]	+ 5.0

*CI* confidence interval; *% pt. diff.* percentage point difference

^†^Calculated from all available data points regardless of the presence of values for variables used in the poststratification process

^*^Only records with available data points for poststratification adjustment could be used to produced adjusted estimates. As such, adjusted estimates are calculated from a truncated data set compared to the data set used to calculate the raw and unweighted estimates

The difference in the RDD and opt-in method of calling respondents was significant at an alpha level of 0.05 for the indicators of households with at least 1 bed net (of any type) and households with at least 1 bed net (of any type) per 2 de facto household population. Assuming a national RDD base rate prevalence of 50% for households with at least 1 bed net, the corresponding prevalence for the national opt-in method is estimated to be 54.1% given an odds ratio of 1.18. Likewise, the indicator of household ownership of at least 1 bed net (of any type) per 2 de facto household population showed a significant difference between the two survey approaches and was associated with an odds ratio of 1.26. This is roughly consistent with a national RDD estimate of 50% prevalence corresponding to a national opt-in estimate of 55.8% prevalence. No significant differences between the two survey approaches were noted when assessing these same two indicators restricted to only ITN availability.

## Discussion

The NMCP and ZAMEP have an established threshold of ≥ 80% de facto household population bed net access which constitutes an acceptable level of coverage. Bed net access levels below this threshold are likely to trigger additional ITN distribution response mechanisms. The results of the current study showed that no region met or exceeded the 80% threshold and in some regions the coverage estimates were rather low (~ 50% in eight regions) indicating an urgent need to ensure that additional ITNs are available. Indeed, the de facto household population access to a bed net ranged from an adjusted percent of 48.1% (Katavi) to 65.5% (Dodoma). Mobile phone survey estimates for the adjusted percent of households with at least one bed net per 2 de facto household population were also relatively low with 46.4% (16 of 28) of regions below 50%. However, nearly one-third (10 of 28; 35.7%) of regions were estimated to have at least 70% coverage of households with at least one bed net. The challenges of achieving and maintaining net access above 80% are significant, given the rates at which bed nets are lost to wear and tear and the challenges of reaching all households with sufficient bed nets when they need them [29, 30].

**Fig. 1:**
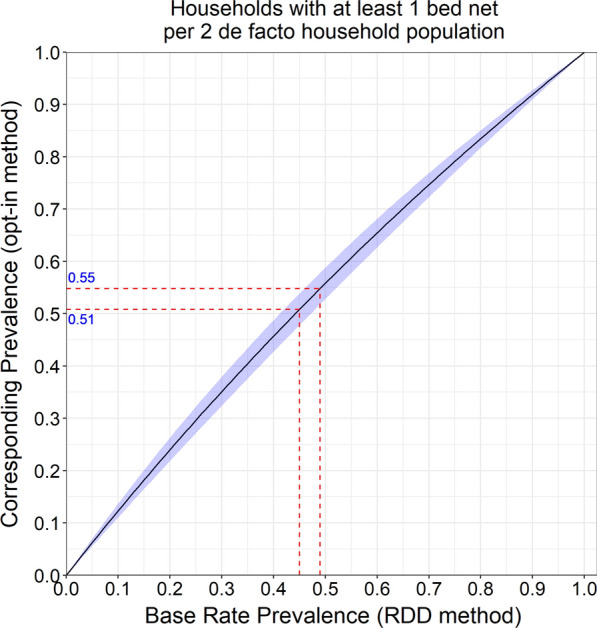
Comparison of mobile phone survey approaches. Using results from the regression output shown in Table 5, it is possible to estimate the difference in indicator values between the two survey approaches (RDD vs. opt-in method) for any base rate prevalence value. Given a strict decision rule to implement a mass distribution campaign once bed net access levels fall below a 50% threshold, a standalone opt-in survey methodology would erroneously trigger a campaign, assuming an RDD gold standard methodology, between prevalence values of 0.51 and 0.55 for households with at least one bed net per 2 de facto household population

The mobile phone survey used for this study employed two different methods to sample respondents. First, a standard RDD approach in which truly random numbers were dialed and secondly, an opt-in-based approach in which a set of pre-identified, opt-in participant phone numbers were sampled for dialing. The estimates produced by both approaches were generally similar. However, the opt-in approach achieved the sample size targets more rapidly and in a wider range of regions of the country including among smaller and more sparsely populated regions. The dual sampling strategy allowed for an assessment of the relative bias of the RDD versus the opt-in approach with respect to household-level bed net coverage indicators. This assessment showed that, while significant, differences between the two survey methodologies were small and would likely not have a programmatically meaningful impact (see caption of Fig. 1). Nationally, the two household-level bed net indicators were estimated to be within 5 percentage points by either sampling strategy. The magnitude of this difference is not likely to greatly alter the decision-making process.

**Table 5:** Presents the results of the secondary objective of the study: the comparison of a true RDD approach to that of an opt-in approach

	Odds Ratio [95% CI]
Household has 1 + bed net (of any type)	*1.18 [1.03, 1.36]
Household has 1 + ITN	1.06 [0.93, 1.21]
Household has 1 + bed net (of any type) per 2 de facto	***1.26 [1.12, 1.43]
Household has 1 + ITN per 2 de facto	1.06 [0.91, 1.25]

RDD vs opt-in method of calling: logistic regression analysis results

Each logistic regression controlled for region, radio ownership, bike ownership, and household size. In each logistic regression equation, the opt-in method was set as the reference category. Observation counts exceeded 4000 for each regression analysis

*RDD* random digit dial; *CI* confidence interval; *ITN* insecticide-treated bed net

^*^ p < 0.05; ** p < 0.01; *** p < 0.001

One explanation for the particularly low bed net coverage estimates noted for several regions may be attributable to the 2020 mass replacement campaign (MRC) conducted for mainland Tanzania. In 10 of the regions selected for the MRC, only certain districts received bed nets. As such, coverage estimates for these 10 regions may be lower than anticipated as this survey includes districts which did not receive replacement bed nets in 2020. For example, only two of Kilimanjaro’s seven districts were targeted for the 2020 MRC, while survey respondents may have resided anywhere within the region, potentially underestimating the overall coverage of Kilimanjaro. Similarly, two of six councils in Njombe and two out of seven councils in Manyara were targeted for the 2020 MRC, the latter of which experienced ongoing distribution at the time of the survey.

Response rates for RDD IVR surveys are typically low, and it is common for breakoffs to occur quickly after successful contacts are made. A previous RDD mobile phone survey was conducted in Tanzania immediately following the 2017 MIS (unpublished data) which achieved a response rate (RR5) of 5.8% compared to the overall RR5 of 1.5% for the current survey. As an additional point of reference, a 2017 RDD IVR study conducted in Ghana reported achieving a response rate of 21% [31]. The low response rates noted for the current study (RR5 of 1.1% and 1.8% for the RDD and opt-in methods, respectively) may be due to an increase in RDD IVR surveys in Tanzania as a precaution against exposing survey enumerators to COVID-19. The overall increase in such mobile phone surveys may lead to respondent fatigue. In addition, in the context of COVID-19, respondents may be preoccupied with other concerns and therefore less likely to respond. Opt-in respondents were collectively expected to demonstrate a higher response rate than participants from the RDD sampling pool, but, surprisingly, their RR5 metric was only slightly higher. The potential benefits from the opt-in approach are shorter call times for those individuals completing the survey due to truncated cascade-style questions on location of residence as well as more willing participants (i.e., a higher response rate) compared to the true RDD approach. Indeed, the opt-in sampling strategy made 31,223 fewer calls than the RDD method but yielded about 100 additional completed surveys.

It is possible that survey participants contacted through the opt-in sampling strategy no longer lived in the region assigned to their record in the opt-in database. In order to simplify the phone interview and to ensure efficient sampling, the already recorded region of residence for these participants was assumed to be accurate and was not asked of them again. While misclassification of region of residence would lead to smoothing of coverage estimates across regions and limit the accuracy of these estimates, the use of more recent opt-in participants should reduce this effect. Note that the mobile phone survey operator managing the opt-in database verifies participant region of residence on at least an annual basis through active solicitation.

In general, non-coverage and non-response biases are potential issues when conducting mobile phone surveys in LMIC. As mobile phone penetration continues to increase across certain LMIC, however, non-coverage bias is gradually reduced [32, 33]. Nevertheless, evidence from recent studies show that MPS tend to oversample male, urban, younger, and better educated respondents – all of whom are generally more likely to own mobile phones in LMIC settings suggesting that non-coverage bias remains an issue [17, 31, 34, 35]. Mobile phone surveys also tend to underrepresent women in Africa, although it remains unclear the extent to which this underrepresentation reflects non-coverage bias or non-response bias [31, 36]. The potential bias in participant type may lead to differential responses to questions concerning the general health and well-being of family members including report on protective measures such as availability and use of bed nets.

The mobile phone survey questionnaire used for this research asked questions related to bed net use for pregnant women and children under five, but the sample size calculation was structured on household ownership of at least one net and did not factor in whether that household would have had members from these higher-risk groups. As such, few data points for these populations were available and calculation of net use indicators was not conducted. Sample size requirements for these target groups are likely to be too large to pragmatically conduct the mobile phone survey given general budget and time considerations.

## Conclusion

Mobile phone surveys can provide rapid estimates of bed net coverage in Tanzania and may serve as a suitable source of information between large-scale household surveys. The veracity of estimates obtained from mobile phone surveys can be validated against household survey estimates, particularly if they are conducted contemporaneously. Based on the results generated by this survey, overall bed net access in the country appears to be lower than target thresholds, with some regions being especially low. The results suggest that bed net distribution is needed in large sections of the country to ensure that access levels remain high enough (above 80%) to permit high levels of bed net use and sustain protection of the population. Lastly, mobile phone survey sampling methodologies based on pre-existing, opt-in respondent lists may be a more efficient and simpler way to collect bed net coverage data compared to RDD methods.

## Supplementary Information

**Additional file 1: **Mobile Phone Survey Questionnaire.

**Additional file 2: **Count of survey responses by indicator and region.

## Data Availability

The datasets used and/or analysed during the current study are available from the corresponding author on reasonable request.

## Abbreviations

DHS: Demographic and Health Surveys
MIS: Malaria Indicator Survey
NMCP: National Malaria Control Programme
ZAMEP: Zanzibar Malaria Elimination Programme
ITN: Insecticide-treated bed net
LMIC: Low- and middle-income countries
IVR: Interactive voice response
RDD: Random digit dial
RBM-MERG: Roll Back Malaria Partnership to End Malaria-Monitoring and Evaluation Reference Group
AAPOR: American Association for Public Opinion Research
MRC: Mass replacement campaign
