# Supplementary material for: Estimation of bed net coverage indicators in Tanzania using mobile phone surveys: a comparison of sampling approaches
Source: Malar J. 2022 Dec 10;21:379. doi: 10.1186/s12936-022-04408-y (PMC9735037; doi:10.1186/s12936-022-04408-y)
Supplement: Supplementary file 1 — Additional file 1: Mobile Phone Survey Questionnaire. [file 12936_2022_4408_MOESM1_ESM.docx]

**Additional File 1. Mobile Phone Survey Questionnaire**

| Question Number | Question |
| --- | --- |
|  | Consent script |
| Consent | Do you agree to participate in this survey and are at least 18 years old? (Multiple Choice Question) |
| Q1 | Region of residence (Cascade-style Question) |
| Q2 | How many people usually live in your house? (Numeric Question) |
| Q3 | How many mosquito nets does your household own? (Numeric Question) |
| Q4 | Did you have enough nets for all your household members to sleep under a net each night? (Multiple Choice Question) |
| Q5 | How many of your mosquito nets were originally or ever treated with insecticide? (Numeric Question) |
| Q6 | How many of your mosquito nets are nets that you purchased? (Numeric Question) |
| Q7 | How many people slept in your household last night? (Numeric Question) |
| Q8 | How many children who are less than five years of age slept in the household in the last night? (Numeric Question) |
| Q9 | How many children less than five years slept under a mosquito net last night? (Numeric Question) |
| Q10 | How many pregnant women slept in your household in the last night? (Numeric Question) |
| Q11 | How many pregnant women slept under a mosquito net last night? (Numeric Question) |
| Q12 | How many adults aged 18 or over usually live in your household? (Numeric Question) |
| Q13 | How many adults aged 18 or above in your household slept under a mosquito net last night? (Numeric Question) |
| Q14 | How many mobile phone SIM cards are owned or regularly used by members of your household? (Multiple Choice Question) |
| Q15 | How many mobile phones are owned or regularly used by members of your household? (Multiple Choice Question) |
| Q16 | Does your household own a bicycle? (Multiple Choice Question) |
| Q17 | Does your household own a television? (Multiple Choice Question) |
| Q18 | Does your household own a radio? (Multiple Choice Question) |
| Q19 | Does your household own a car? (Multiple Choice Question) |
| Q20 | Did the head of household complete primary school? (Multiple Choice Question) |
| Q21 | Would you like to participate in a future survey? (Multiple Choice Question) |
