# Supplementary material for: Estimation of bed net coverage indicators in Tanzania using mobile phone surveys: a comparison of sampling approaches
Source: Malar J. 2022 Dec 10;21:379. doi: 10.1186/s12936-022-04408-y (PMC9735037; doi:10.1186/s12936-022-04408-y)
Supplement: Supplementary file 2 — Additional file 2: Count of survey responses by indicator and region. [file 12936_2022_4408_MOESM2_ESM.docx]

**Additional File 2. Count of survey responses by indicator and region**

|  | Household has 1+ net | Household has 1+ net per 2 de facto population | De facto population access to a net | Nets self-reported as purchased | Nets self-reported as originally or ever treated with insecticide |
| --- | --- | --- | --- | --- | --- |
| Arusha | 611 | 468 | 468 | 405 | 482 |
| Dar es salaam | 578 | 421 | 421 | 368 | 436 |
| Dodoma | 523 | 391 | 391 | 353 | 389 |
| Geita | 511 | 350 | 350 | 363 | 398 |
| Iringa | 555 | 436 | 436 | 371 | 401 |
| Kagera | 534 | 402 | 402 | 399 | 415 |
| Katavi | 295 | *185 | *185 | *200 | *228 |
| Kigoma | 430 | 275 | 275 | 292 | 323 |
| Kilimanjaro | 536 | 404 | 404 | 380 | 422 |
| Lindi | 312 | *203 | *203 | *206 | 239 |
| Manyara | 367 | 252 | 252 | *247 | 279 |
| Mara | 453 | 348 | 348 | 326 | 364 |
| Mbeya | 518 | 403 | 403 | 345 | 367 |
| Morogoro | 520 | 359 | 359 | 328 | 406 |
| Mtwara | 286 | *191 | *191 | *173 | *222 |
| Mwanza | 620 | 443 | 443 | 447 | 474 |
| Njombe | 382 | 304 | 304 | 278 | 310 |
| Pemba | *213 | *121 | *121 | *133 | *166 |
| Pwani | 379 | 274 | 274 | *249 | 288 |
| Rukwa | 332 | *217 | *217 | *214 | *239 |
| Ruvuma | 473 | 348 | 348 | 325 | 361 |
| Shinyanga | 483 | 341 | 341 | 329 | 396 |
| Simiyu | 388 | 272 | 272 | 261 | 315 |
| Singida | 510 | 353 | 353 | 329 | 363 |
| Songwe | 382 | 260 | 260 | 256 | 288 |
| Tabora | 549 | 371 | 371 | 369 | 427 |
| Tanga | 507 | 360 | 360 | 316 | 375 |
| Unguja | 693 | 395 | 395 | 408 | 522 |

*Below desired target of 250 observations.
